# Gene probing reveals the widespread distribution, diversity and abundance of isoprene-degrading bacteria in the environment

**DOI:** 10.1186/s40168-018-0607-0

**Published:** 2018-12-07

**Authors:** Ornella Carrión, Nasmille L. Larke-Mejía, Lisa Gibson, Muhammad Farhan Ul Haque, Javier Ramiro-García, Terry J. McGenity, J. Colin Murrell

**Affiliations:** 1 School of Environmental Sciences, University of East Anglia, Norwich Research Park, Norwich, NR4 7TJ UK; 2 Luxembourg Centre for Systems Biomedicine, University of Luxembourg, Esch-sur-Alzette, Luxembourg; 3 School of Biological Sciences, University of Essex, Colchester, UK

**Keywords:** Isoprene, Climate, Isoprene monooxygenase, *isoA*, Gene probes

## Abstract

**Background:**

Approximately 500 Tg of isoprene are emitted to the atmosphere annually, an amount similar to that of methane, and despite its significant effects on the climate, very little is known about the biological degradation of isoprene in the environment. Isolation and characterisation of isoprene degraders at the molecular level has allowed the development of probes targeting *isoA* encoding the α-subunit of the isoprene monooxygenase. This enzyme belongs to the soluble diiron centre monooxygenase family and catalyses the first step in the isoprene degradation pathway. The use of probes targeting key metabolic genes is a successful approach in molecular ecology to study specific groups of bacteria in complex environments. Here, we developed and tested a novel *isoA* PCR primer set to study the distribution, abundance, and diversity of isoprene degraders in a wide range of environments.

**Results:**

The new *isoA* probes specifically amplified *isoA* genes from taxonomically diverse isoprene-degrading bacteria including members of the genera *Rhodococcus*, *Variovorax*, and *Sphingopyxis*. There was no cross-reactivity with genes encoding related oxygenases from non-isoprene degraders. Sequencing of *isoA* amplicons from DNA extracted from environmental samples enriched with isoprene revealed that most environments tested harboured a considerable variety of *isoA* sequences, with poplar leaf enrichments containing more phylogenetically diverse *isoA* genes. Quantification by qPCR using these *isoA* probes revealed that isoprene degraders are widespread in the phyllosphere, terrestrial, freshwater and marine environments. Specifically, soils in the vicinity of high isoprene-emitting trees contained the highest number of isoprene-degrading bacteria.

**Conclusion:**

This study provides the molecular ecology tools to broaden our knowledge of the distribution, abundance and diversity of isoprene degraders in the environment, which is a fundamental step necessary to assess the impact that microbes have in mitigating the effects of this important climate-active gas.

**Electronic supplementary material:**

The online version of this article (10.1186/s40168-018-0607-0) contains supplementary material, which is available to authorized users.

## Background

Isoprene (2-methyl-1, 3-butadiene) comprises approximately one third of the total volatile organic compounds (VOC) emitted to the atmosphere, an amount that is approximately equal to emissions of methane [1, 2]. Although isoprene has a short lifetime in the atmosphere (in the order of hours) due to rapid photochemical oxidation [1], it has a significant impact on atmospheric chemistry and hence climate [3]. In unpolluted environments with low levels of nitrogen oxides, isoprene reacts with hydroxyl radicals, thus reducing the oxidising capacity of the atmosphere [1]. This, in turn, prolongs the lifetime of greenhouse gases such as methane and enhances global warming [4, 5]. In polluted environments, nitrogen oxides are typically present at high concentrations and react with isoprene, leading to the formation of tropospheric ozone [1], which is a greenhouse gas with important negative effects on plant and animal health [6]. Conversely, atmospheric oxidation of isoprene results in the formation of secondary organic aerosols and cloud condensation nuclei, which in turn promotes global cooling [7, 8]. The vast majority of isoprene emitted to the atmosphere is produced by terrestrial plants (~ 500 Tg year^−1^) [2, 9], with small contributions from marine algae (0.1–12 Tg year^−1^) and minor contributions from bacteria, fungi and animals [10–17]. Isoprene is also produced industrially (~ 0.8 Tg year^−1^), where it is used primarily to synthesise polyisoprene rubber [18]. In plants, isoprene is synthesised in the chloroplast from dimethylallyl diphosphate (DMAPP), an intermediate of isoprenoid biosynthesis, in a reaction mediated by isoprene synthase [19]. It has been shown that isoprene protects plants against heat and oxidative stress [9, 20] and it has also been suggested that it might have a role in plant-insect interactions [21] and plant energy dynamics [22]. However, not all plants produce isoprene, with both high and low emitters being observed even among closely related species [23–25].

Although atmospheric levels of isoprene are low (1–4 ppb) [26], due to its high reactivity, concentrations are significantly higher (up to 36 ppb) at ground level in high isoprene-emitting forests [27]. Closed chambers and continuous-flow experiments have shown that soils can act as a biological sink for isoprene at environmentally relevant concentrations [28–30]. These studies confirmed the potential for soil microbes to consume isoprene released locally in soils as well as from the atmosphere. In fact, bacteria that grow on isoprene as sole carbon and energy source have been isolated from soils, leaves and coastal/marine environments [31–37]. These isolates are mainly Actinobacteria, although recently more Alpha- and Betaproteobacteria strains such as *Sphingopyxis* sp. OPL5 and *Variovorax* sp. WS11 have also been isolated [38]. All known isoprene degraders contain six genes (*isoABCDEF*) encoding the isoprene monooxygenase (IsoMO) that catalyses the first step of the isoprene degradation pathway. Four additional genes, *isoGHIJ*, are located immediately upstream (5′) of the IsoMO structural genes and encode enzymes involved in the subsequent steps in isoprene catabolism [32, 33, 39, 40]. The IsoMO is a four-component soluble diiron monooxygenase (SDIMO) composed of a dimeric hydroxylase, a NAD(P)H oxidoreductase, a coupling protein and a Rieske-type ferredoxin. Other members of the SDIMO family include the soluble methane monooxygenase (sMMO), alkene monooxygenases, phenol hydroxylases and aromatic monooxygenases, which are key enzymes in the bacterial oxidation of hydrocarbons and have biotechnological applications [41, 42]. In addition, structural and genetic analyses have revealed that the hydroxylase α-subunit of SDIMOs contains a carboxylated-bridge diiron centre in a distinctive 4-helix bundle structure at the active site (reviewed in [42]).

**Fig. 1:**
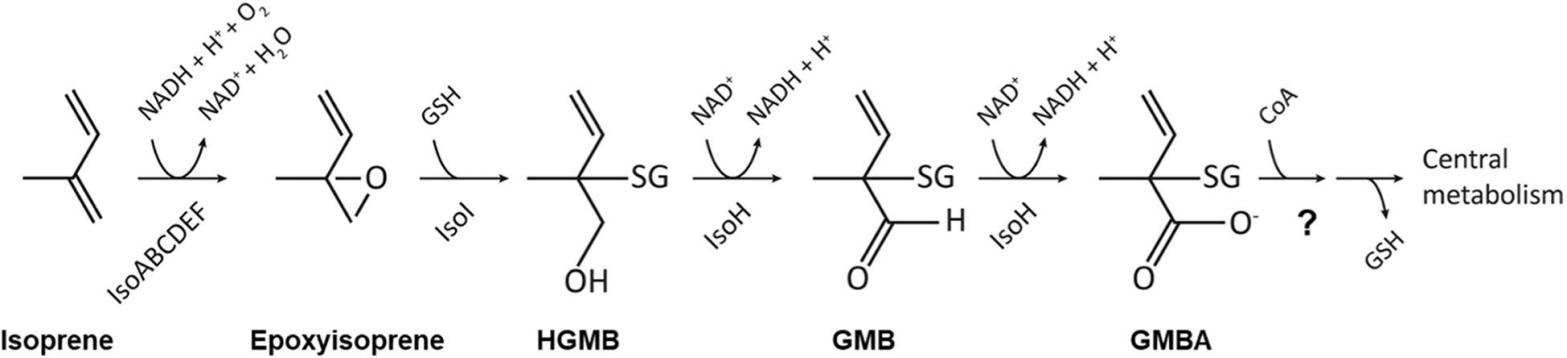
Isoprene degradation pathway. Enzymes: IsoABCDEF, isoprene monooxygenase; IsoI, glutathione-*S*-transferase; IsoH, dehydrogenase. *HGMB* 1-hydroxy-2-glutathionyl-2-methyl-3-butene, *GMB* 2-glutathionyl-2-methyl-3-butenal, *GMBA* 2-glutathionyl-2-methyl-3-butenoic acid, *SG* glutathione, *GSH* reduced glutathione. The question mark indicates uncertainty in the details of the catabolic pathway from GMBA

The IsoMO catalyses the initial oxidation of isoprene to 1,2-epoxyisoprene (Fig. 1). The epoxide is converted to 1-hydroxyl-2-glutathionyl-2-methyl-3-butene (HGMB) by a glutathione *S*-transferase (IsoI) and then by a dehydrogenase (IsoH) to 2-glutathionyl-2-methyl-3-butenoate (GMBA) [33]. The fate of GMBA is uncertain. It is assumed that subsequent removal of glutathione and β-oxidation of these intermediates enable isoprene degraders to grow on isoprene as a carbon source but the final steps in the catabolism of isoprene remain to be elucidated.

The identity and availability of genes essential for isoprene metabolism provide tools for cultivation-independent studies to assess the distribution, diversity and activity of isoprene degraders in the environment. Gene probes are important molecular ecology tools to study functional groups of interest in complex environments. For example, primers targeting genes encoding key subunits of particulate and soluble methane monooxygenase (*pmoA* and *mmoX*, respectively) have been used to extend our knowledge of the diversity and abundance of methane-oxidising bacteria in many environments [43–46]. The *isoA* gene, encoding the α-subunit harbouring the active site of IsoMO, is highly conserved in all isoprene degraders studied and is a suitable marker gene for isoprene degradation [36]. Primers targeting *isoA*, tested negative with genes encoding the corresponding active site of SDIMOs from non-isoprene degraders, but amplified *isoA* from extant isoprene-degrading bacteria and from a range of environmental samples enriched with isoprene, generating sequences which were > 86% identical to those of bona-fide isoprene degraders [36]. However, the increasing number and variety of isoprene degraders has revealed a higher diversity of *isoA* sequences, which emphasises the need to refine *isoA* primers to cover all the isoprene-degrading strains characterised to date. Here, we designed new *isoA* primers that amplified *isoA* genes from all extant isoprene degraders, but did not amplify *isoA* homologues of related enzymes from non-isoprene degraders. These new *isoA* probes were then used to investigate the distribution, diversity and abundance of isoprene degraders in phyllosphere, soils, freshwater and marine environments to better understand their role in the isoprene biogeochemical cycle.

## Results and discussion

### Design and validation of *isoA* primers

In order to cover the diversity of all well-characterised isoprene degraders, we designed new probes targeting *isoA*, as this gene encodes the α-subunit containing the active site of the IsoMO and is an excellent marker gene for isoprene degradation [36]. We aligned the *isoA* genes from 38 bona-fide isoprene degraders available from Genbank and strains recently isolated by our group (Additional file 1: Table S1). Microorganisms included in the analysis belonged to the classes Actinobacteria (e.g. *Gordonia*, *Mycobacterium*, *Rhodococcus*), Alphaproteobacteria (e.g. S*phingopyxis*) and Betaproteobacteria (e.g. *Variovorax*). Eighteen *isoA* sequences detected in the metagenomes from isoprene enrichments of willow soil, willow leaves and poplar leaves (unpublished data) were added to the *isoA* database to design the new probes. These metagenome-derived sequences had a minimum query coverage of 98% and an identity of ≥ 85% at the derived amino acid level to ratified IsoA sequences. Finally, genes encoding the α-subunit of other SDIMOs such as sMMO, alkene monooxygenase or toluene monooxygenase from non-isoprene-degrading microorganisms (Additional file 1: Table S1) were also included in the alignment to guide the specific amplification of *isoA* genes by the new probes. Conserved positions within the *isoA* gene were identified and various sets of primers spanning different regions were manually designed (Additional file 1: Table S2; Figure S1). Eleven different combinations of primers were initially tested (Additional file 1: Table S3), including isoA14F and isoA1019R, which have been previously investigated [38]. In this preliminary validation of the *isoA* probes, *Rhodococcus* sp. AD45 and *Variovorax* sp. WS9 were selected as representative Gram-positive and Gram-negative isoprene-degrading bacteria. *Xanthobacter autotrophicus* Py2 was chosen as a negative control since the α-subunit of its alkene monooxygenase is closely related to IsoA (70% amino acid identity to IsoA from *Rhodococcus* sp. AD45).

Five out of the 11 combinations of primers tested yielded a PCR product of the expected size from the positive control strains, but there was no amplification from *Xanthobacter autotrophicus* Py2 (Additional file 1: Figure S2). Combinations isoA136F + isoA1019R, isoA300F + isoA1019R, isoA379F + isoA862R and isoA379F + isoA1019R also generated non-specific amplification products (Additional file 1: Figure S2). However, the combination of primers isoA14F and isoA511R, which spans the first iron centre of the IsoMO α-subunit (Additional file 1: Figure S1), yielded a specific PCR product of 497 bp. Therefore, we selected primers isoA14F and isoA511R for further validation on genomic DNA from additional isoprene-degrading and non-degrading isolates (Additional file 1: Table S4). A specific amplification product of the expected size was obtained for all 30 positive control strains used in this study. To check the cross-reactivity of the primers isoA14F and isoA511R, we used as negative controls 12 non-isoprene-utilising strains with related oxygenases to IsoMO that grow on alkanes, alkenes or aromatic compounds. Examples include bacteria containing sMMO (*Methylococcus capsulatus* Bath), toluene monooxygenase (*Pseudomonas mendocina* KR1) or alkene monooxygenase (*Rhodococcus rhodochrous* B276). We also studied as negative controls two strains that belong to the same genera as the bona-fide isoprene degraders *Rhodococcus* (*Rhodococcus opacus* DSM 1069) and *Variovorax* (*Variovorax paradoxus* EPS), but do not oxidise isoprene (Additional file 1: Table S4). No PCR products were obtained with the primers isoA14F and isoA511R with template DNA from any of the negative control strains. To check that the lack of amplification was not due to the quality of the DNA, a 16S rRNA gene PCR was performed and strong amplification products were obtained with DNA from all 14 negative control strains (data not shown). Therefore, we conclude that the primers isoA14F and isoA511R are specific for *isoA* encoding the α-subunit of the IsoMO and ratify that *isoA* is an excellent marker gene to determine if isoprene-degrading bacteria contain IsoMO.

### Diversity of *isoA* genes in environmental samples

To test the specificity of the new *isoA* primer set and to investigate the diversity of *isoA* genes, and thus isoprene degraders in various environments, we enriched 11 samples from phyllosphere, soils, freshwater and marine environments with isoprene (Additional file 1: Table S5). DNA extracted from these enrichments was subjected to PCR amplification with the *isoA* primer set isoA14F and isoA511R. A single PCR product of the correct size (497 bp) was obtained with all the enrichments. To confirm that these PCR products contained only *isoA* genes, before committing to high-throughput sequencing of *isoA* amplicons, 9 out of the 11 enriched environmental samples were selected to construct *isoA* libraries from purified PCR amplicons. Sixty-nine clones from these *isoA* libraries were sequenced (Additional file 1: Table S5). Bioinformatic analysis using BLASTx [47] confirmed all sequences as *isoA* genes as they had an identity of 84–100% at the derived amino acid level to IsoA from bona-fide isoprene degraders and were ≤ 70% identical to other α-subunits of closely related SDIMOs that are not IsoMO such as the alkene monooxygenase from *Xanthobacter autotrophicus* Py2.

**Fig. 2:**
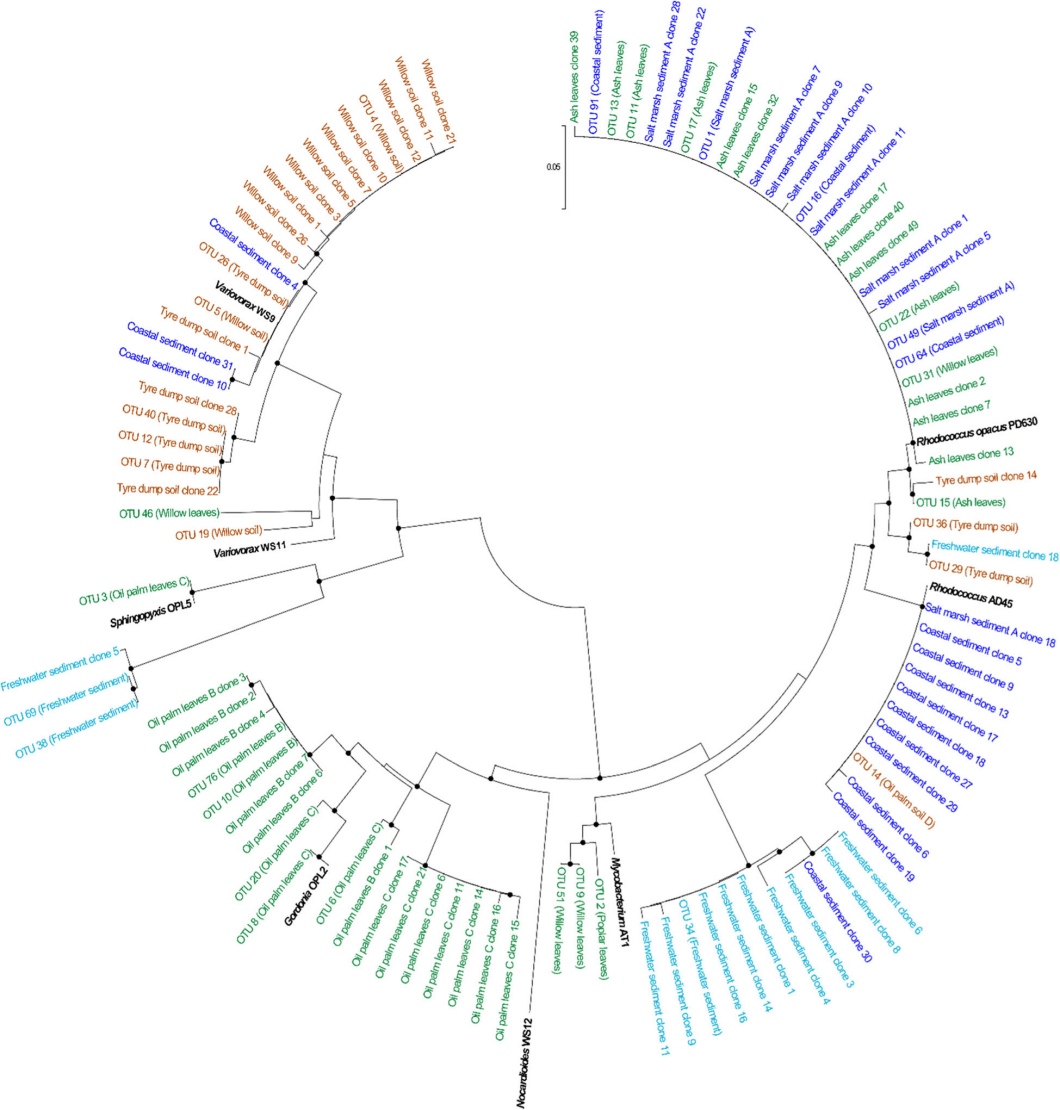
Phylogenetic tree of IsoA sequences retrieved from enriched environmental samples. Sequences in black represent IsoA sequences from bona-fide isoprene degraders. Sequences obtained from clone libraries and amplicon sequencing of leaf samples are represented in green, soils in brown, freshwater environments in light blue and marine sediments in dark blue. Environments where a particular OTU is abundant are shown in brackets. Only OTUs with ≥ 2% abundance in at least one of the samples are represented. The tree was drawn in Mega7 [82] using the neighbour-joining method and the Jones-Taylor-Thornton model. Scale bar indicates 0.05 substitutions per site. Bootstrap values ≥ 50% (based on 1000 replicates) are represented with dots at branch points

The diversity of *isoA* sequences in DNA extracted from enriched samples from leaves varied according to the type of tree sampled. For example, *isoA* genes from ash leaf enrichments were similar to *isoA* from *Rhodococcus*, whereas *isoA* genes from oil palm leaf samples were phylogenetically close to *isoA* from *Gordonia* (Fig. 2). It was not surprising to find *Rhodococcus isoA* homologues in leaf enrichments, as several isoprene-degrading bacteria from this genus have been obtained from poplar, willow, oil palm and horse chestnut leaves [35, 36, 38]. In addition, *Gordonia* strains able to grow on isoprene have been previously isolated from an estuarine environment and oil palm leaves [31, 38].

Most of the *isoA* sequences retrieved from soils were phylogenetically close to *isoA* from *Variovorax*, although *Rhodococcus isoA* homologues were also found in DNA from tyre dump soil enrichments (i.e. tyre dump soil clone 14; Fig. 2). *Variovorax* strains are common inhabitants of soil and water [48–50], and are frequently associated with the phyllosphere [51, 52] and rhizosphere [53, 54] of plants. Indeed, some species of this genus, such as *Variovorax paradoxus*, are considered plant growth-promoting rhizobacteria that exert beneficial effects on plant growth [54]. *Variovorax* species are metabolically versatile bacteria that can degrade a wide range of natural and xenobiotic compounds such as alkanesulfonates, polychlorinated biphenyls or trichloroethylene [55]. Larke-Mejía [38] has shown that two novel *Variovorax* strains, isolated from soil surrounding a willow tree can grow on isoprene. The presence of *Rhodococcus isoA* homologues in soil collected from a tyre dump site is consistent with the recent isolation of isoprene-degrading *Rhodococcus* strains from these samples [38].

In freshwater sediment enrichments, the predominant *isoA* sequences were phylogenetically closer to *isoA* from *Rhodococcus*, though sequences similar to *isoA* from *Sphingopyxis* were also present (i.e. freshwater sediment clone 5; Fig. 2). In fact, *Rhodococcus* sp. AD45, the most well-characterised isoprene degrader, was isolated from freshwater sediment [32]. *Sphingopyxis* is a genus commonly associated with the phyllosphere [51, 52], although *Sphingopyxis* species have also been isolated from soils, freshwater and seawater samples [56–59]. It was not until the isolation of *Sphingopyxis* sp. OPL5 [38], however, that members of this genus were shown to metabolise isoprene.

Finally, in salt marsh sediment enrichments, only *isoA* sequences from *Rhodococcus* were found, whereas enrichments with coastal sediment yielded more diverse *isoA* sequences, with both *Rhodococcus* and *Variovorax isoA*-like genes being obtained from the clone libraries (i.e. coastal sediment clones 4 and 5; Fig. 2). Indeed, the presence of *Rhodococcus isoA* homologues in marine environments has been confirmed with the recent isolation of two isoprene-degrading *Rhodococcus* strains from salt marsh and coastal sediments (unpublished data).

**Fig. 3:**
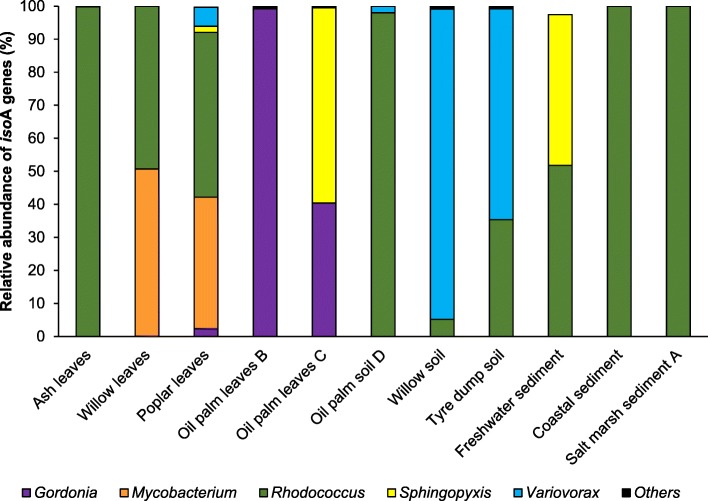
Relative abundance and diversity of *isoA* genes in enriched environmental samples revealed by amplicon sequencing. *isoA* amplicon yielded an average of 12,453 quality-filtered reads per sample. After analysis with the DADA2 pipeline [60] a final set of 134 unique OTUs was obtained (Additional file 2: Table S6). Only OTUs with ≥ 1% abundance in at least one of the samples are represented. For relative abundance of individual OTUs in each enriched environmental sample, see Additional file 1: Figure S3. For α-diversity of enriched environmental samples estimated using Shannon index, see Additional file 1: Table S7

Since the sequences obtained from the clone libraries were specific to *isoA* and showed variability within and across the different ecosystems studied, we explored in more detail the diversity of *isoA* genes in the environment using high-throughput sequencing. Purified *isoA* PCR products from DNA isolated from 11 enriched environmental samples were sequenced using Illumina MiSeq technology (Additional file 1: Table S5). *isoA* amplicon sequencing yielded a total of 136,986 quality-filtered sequences with an average of 12,453 reads per sample. These *isoA* sequences, when analysed by the DADA2 pipeline [60], grouped into 136 unique operational taxonomic units (OTUs) that were manually checked by BLASTx (see Methods). Two OTUs, representing 0.3% and 2.5% of the sequences from DNA extracted from enriched poplar leaves and freshwater sediment, respectively, were discarded for the analysis as they had hits to proteins not related to IsoA or other SDIMO α-subunits. Therefore, a final set of 134 OTUs was used for downstream analysis (Fig. 3; Additional file 2: Table S6).

The *isoA* amplicon sequencing analysis revealed that the phyllosphere yielded most variability between samples when compared to other environments (Fig. 3). For example, 99.8% of the *isoA* sequences from ash leaf enrichments were similar to *isoA* from *Rhodococcus*. *isoA* genes similar to those of *Gordonia* and *Mycobacterium* were also found, although at low relative abundance (< 1%). Conversely, DNA from oil palm leaf enrichments yielded predominantly *Gordonia* and *Sphingopyxis isoA* homologues, even though *isoA* genes from *Rhodococcus* and *Mycobacterium* were also present (< 1%). Indeed, we have recently isolated isoprene-degrading *Gordonia* and *Sphingopyxis* strains from oil palm leaves [38]. The predominant sequences in willow leaf enrichments were phylogenetically close to *isoA* from *Mycobacterium* (50.7%) and *Rhodoccocus* (49.3%). Finally, poplar leaf enrichments yielded more phylogenetically diverse *isoA* genes, with homologues to *isoA* from *Gordonia* (2.3%), *Mycobacterium* (40%), *Rhodococcus* (50%), *Sphingopyxis* (1.9%), and *Variovorax* (5.8%, Fig. 3). Although these genera are common inhabitants of the phyllosphere and soils [50, 51, 54, 61], no *Variovorax* or *Mycobacterium* strains from leaves able to metabolise isoprene have been reported so far.

The most abundant *isoA* sequences identified in willow, oil palm and tyre dump soil enrichments were similar to *Rhodococcus* and *Variovorax isoA* genes (Fig. 3). *isoA* sequences from *Mycobacterium*, *Nocardioides* and *Sphingopyxis* were also detected in willow soil (< 1%). Sequences similar to *isoA* from *Gordonia*, *Mycobacterium* and *Sphingopyxis* were present at low relative abundance (< 1%) in DNA extracted from tyre dump soil. These results are consistent with the isolation of *Nocardioides*, *Rhodococcus* and *Variovorax* isoprene-degrading strains from willow and tyre dump soils [35, 38].

Finally, *isoA* homologues from *Sphingopyxis* (46.8%) and *Rhodococcus* (53.2%) dominated DNA from freshwater sediment enrichments, whereas *isoA* sequences retrieved from coastal and salt marsh enriched samples had highest identity to *isoA* from *Rhodococcus* (Fig. 3).

In addition, several sequences were distinct from those of *isoA* of bona-fide isoprene degraders, indicating that there is likely novel diversity of isoprene-utilising bacteria yet to be discovered. For example, most of the *isoA* clones and OTUs originating from enriched freshwater sediment samples (i.e. freshwater sediment clones 5 and 8; OTUs 34 and 38) occupied a distinct position in the IsoA phylogenetic tree as shown in Fig. 2. BLASTx analysis of these sequences revealed that they had 83–91% identity at the derived amino acid level to IsoA from extant isoprene degraders of the genera *Rhodococcus* and *Sphingopyxis*, suggesting that this environment harbours novel isoprene-utilising strains. The sequencing information obtained using the new *isoA* probes isoA14F and isoA511R can now be used to design targeted enrichment and isolation strategies to isolate novel species of isoprene degraders from various environments and expand the diversity of existent isoprene-degrading bacteria.

### Distribution and abundance of isoprene degraders in the environment

*isoA* primer set isoA14F and isoA511R was used to study the distribution and abundance of *isoA*-containing bacteria using qPCR. *isoA* primers were first optimised and validated on DNA extracted from environmental samples enriched with isoprene (Additional file 1: Figure S5). Subsequently, clone libraries from qPCR products obtained from both enriched and natural (non-enriched) environmental samples were constructed to ensure that *isoA* qPCR products were absolutely specific for *isoA*. Fifty-two clones from these *isoA* libraries were sequenced. All sequences had 89–100% identity at the derived amino acid level to IsoA from bona-fide isoprene degraders and were ≤ 70% identical to other α-subunits of closely related SDIMOs that are not IsoMO such as the alkene monooxygenase from *Xanthobacter autotrophicus* Py2.

**Fig. 4:**
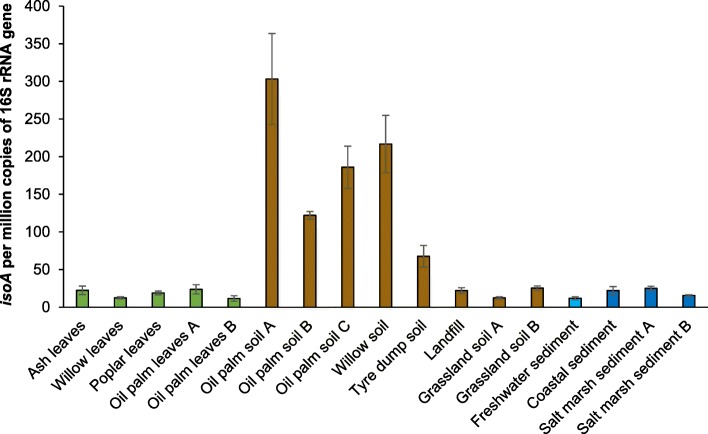
Relative abundance of isoprene degraders in natural (non-enriched) environmental samples estimated by qPCR. *isoA* copies are normalised to the 16S rRNA gene copy number in each sample. Results shown are the average of triplicate samples. Error bars represent standard deviations. Leaf samples are represented in green, soils in brown, freshwater environments in light blue and marine sediments in dark blue

After qPCR assay validation, the abundance of isoprene degraders across a wide range of natural samples, including leaves, soils, freshwater and marine sediments was studied. Interestingly, leaves from high isoprene-emitting trees such as willow, poplar and oil palm [62, 63] contained similar *isoA* numbers as those from an ash tree, a low isoprene emitter (between 11.6 ± 3.6 and 23.7 ± 6.1 *isoA* genes per million copies of 16S rRNA gene in DNA extracted from high isoprene-emitting trees, versus 22.4 ± 5.6 *isoA* genes per million copies of 16S rRNA gene in DNA from ash leaves; Fig. 4). However, a larger number of environmental samples will be required to confirm statistically that there are no significant differences in the abundance of isoprene degraders between high and low-emitting trees. Moreover, soils sampled in the vicinity of high isoprene-emitting trees yielded the highest numbers of isoprene degraders (with values ranging from 122.2 ± 5.0 to 303.3 ± 60.3 *isoA* genes per million copies of 16S rRNA gene; Fig. 4). The fact that there were 10-fold more isoprene degraders in soils than in the leaves of the same trees could be explained by the flux of bacteria from the phyllosphere to the pedosphere during rainfall, which has been estimated to be up to 1.5 × 10^16^ cells ha^−1^ year^−1^ in a subtropical oak-cedar forest [64]. It was not surprising to see relatively high numbers of *isoA* genes (67.7 ± 14.4 *isoA* genes per million copies of 16S rRNA gene) in soil sampled from a tyre dump, since tyres mainly consist of polyisoprene rubber [65, 66]. Although several bacteria and fungi can degrade polyisoprene rubber [67, 68], some studies have suggested that actinomycetes are the key microorganisms carrying out this process [69–72]. The cleavage of polyisoprene rubber by Lcp and RoxA oxygenases results in the production of oligoisoprene molecules [73] that could potentially be used by the isoprene degraders for growth. Indeed, rubber-contaminated soils have yielded isoprene-utilising bacteria from different genera, including *Rhodococcus* [38, 74], although these strains were not characterised in detail. Grassland and landfill soils also contained isoprene degraders, although at lower relative abundance (from 12.4 ± 1.3 to 25.4 ± 2.8 *isoA* genes per million copies of 16S rRNA gene) than soils surrounded by high isoprene-emitting trees, as anticipated (Fig. 4). The presence of isoprene-degrading microorganisms was also studied in freshwater and marine natural samples. Surprisingly, these samples yielded similar copy numbers of *isoA* genes (ranging from 12.0 ± 1.9 to 25.1 ± 2.8 *isoA* copies per million copies of 16S rRNA gene) as leaves (Fig. 4). This was unexpected since isoprene emissions from marine environments (0.1–12 Tg year^−1^) [2, 11, 16] are very much lower than from terrestrial plants (500 Tg year^−1^) [2, 9]. In marine environments, isoprene is synthesised by phytoplankton, heterotrophic bacteria and seaweeds [16]. However, only a few studies have directly measured the isoprene concentration in the euphotic zones of the oceans and the exact mechanism behind marine isoprene production is still unknown [75]. Another poorly understood aspect of the marine isoprene cycle is the role of microbial degradation. Ocean depth profiles of isoprene concentrations have suggested that isoprene is biologically consumed [75–78]. Indeed, Acuña Alvarez et al. [31] and Johnston et al. [37] found isoprene-degrading bacteria in estuarine and marine water samples, most of which were Actinobacteria. More importantly, Acuña Alvarez et al. [31] also showed that isoprene-utilising bacteria degraded isoprene from the headspace of microalgae cultures at environmentally relevant concentrations. A recent study by Steinke et al. [17] has reported that freshwater lakes also emit isoprene to the atmosphere. Therefore, additional laboratory experiments and field studies as well as more accurate models are required to better understand the isoprene cycle in marine and freshwater environments.

## Conclusions

Although we cannot exclude the possibility that *isoA* genes from novel uncultivated isoprene-degrading bacteria have been missed, or that other pathways of isoprene metabolism exist, new probes targeting the *isoA* gene encoding the active site of the IsoMO have proven to be a successful tool to study the diversity, distribution and abundance of isoprene degraders in a wide range of environments. This now facilitates the development of targeted strategies to isolate novel genera of isoprene degraders, monitor them under natural conditions and to determine how isoprene degradation is regulated in the environment. This study provides molecular probes to investigate the significance of the isoprene biological sink and how bacteria might mitigate the effect on the atmosphere of this abundant climate-active gas.

## Methods

### Enrichments of environmental samples with isoprene

To study the distribution, abundance and diversity of isoprene degraders in the environment, a wide range of terrestrial, phyllosphere, freshwater and marine samples were collected (Additional file 1: Table S5). Terrestrial samples comprised soils from the vicinity of high isoprene-emitting trees (oil palm and willow) and soils without nearby high isoprene-emitting vegetation (grassland and landfill soils). Soil from a tyre dump was also studied since several isoprene-degrading strains have been isolated from rubber-contaminated soils [38, 74]. Samples from the phyllosphere included leaves from high isoprene-producing trees as for example, willow, poplar and oil palm, and low emitters such as ash trees. Freshwater samples were collected from a local lake. Samples from marine environments consisted of coastal and salt marsh sediments (Additional file 1: Table S5).

In the case of soils, freshwater and marine sediments, only the top 3 cm were collected after removing vegetation or macroscopic algae. Further, 1 g of material was used to set up microcosm enrichments in 120 ml sealed vials containing 10 ml of water for soils, Ewers medium [79] for freshwater sediments or marine basal medium (MBM) [80] for marine samples. Salinity of MBM was adjusted to 20 practical salinity units (PSU) in coastal sediment samples and to 35 PSU in salt marsh sediments to mimic natural conditions.

The surface of 3–10 leaves from each tree (depending on leaf size) was sampled by washing leaves with Ewers medium or using cotton swabs. For leaf washings, leaves were immersed in 50 ml of Ewers medium in 250 ml conical flasks and submerged in a water bath for sonication (5 min, 50 kHz; Mettler ME2), followed by shaking on an orbital shaker (1 h, 150 rpm) to detach microbial cells from the surface of the leaves. Leaves were then removed from the flasks and medium was centrifuged (5000×*g*, 20 min) to separate cells and particulate material from the supernatant. Supernatant was filtered through CellTrap CT402LL001N00 filters (Mem-Teq). Pellets of cells and particulate material were combined with the filtrated supernatant, resuspended in 10 ml of fresh minimal medium and transferred to 120 ml sealed vials. When leaf surfaces were sampled with cotton swabs (ash leaves and oil palm leaves C), the swabs were placed in 250 ml conical flasks containing 50 ml of Ewers medium and sonicated in a water bath as above. After shaking in an orbital shaker for 1 h at 150 rpm, cotton swabs were removed and medium was aliquoted into 120 ml sealed vials.

All enrichments were set up in triplicate and incubated at 25 °C with 25 ppm isoprene, except for oil palm leaf samples from Malaysia, which were incubated at 30 °C with 50 ppm of isoprene. Consumption of isoprene was monitored daily by gas-chromatography as described in [34]. When isoprene was depleted in the headspace, samples were spiked again with 25 ppm of isoprene or 50 ppm in the case of oil palm leaf samples from Malaysia. Enrichments were subcultured at 2-week intervals three times by making 1/10 dilutions of the samples in fresh medium.

### DNA extraction from bacterial strains and environmental samples

Genomic DNA from positive and negative control strains was extracted from cultures grown in rich media using the Wizard Genomic DNA Purification Kit (Promega), according to the manufacturer’s instructions.

To extract DNA from environmental samples, the FastDNA™ SPIN kit for Soil (MP Biomedicals) was used following the protocol described by the manufacturer.

### Amplification of *isoA* genes

Fifty nanograms of genomic DNA or 20 ng to 1 μg of environmental DNA were used as a template in a 50 μl PCR reaction containing 4 μM of isoA14F (5′-GVGACGAYTGGTAYGACA-3′) and isoA511R (5′-TCGTCRAAGAARTTCTTBAC-3′) primers. The PCR program consisted of an initial step of 94 °C for 2 min, followed by 31 cycles of 95 °C for 15 s, 54 °C for 30 s, 72 °C for 1 min and a final extension step of 72 °C for 7 min. In the case of freshwater and marine DNA samples, 40 cycles were carried out to obtain an amplicon visible on an agarose gel stained with ethidium bromide.

### Clone libraries

*isoA* PCR products from environmental samples were purified using the NucleoSpin gel and PCR Clean-up kit (Macherey-Nagel) and cloned into the pGEM®-T easy vector system (Promega) following the manufacturer’s instructions prior to transformation into *Escherichia coli* TOP10 cells. Positive clones were screened by PCR using the M13F and M13R primers. Clones yielding a PCR product were sent for sequencing using M13 primers.

### *isoA* amplicon sequencing

Duplicate PCR products from each environmental sample were pooled before DNA purification using the NucleoSpin gel and PCR Clean-up kit (Macherey-Nagel). The quality of DNA was assessed by gel electrophoresis and the Qubit dsDNA High Sensitivity Assay Kit (ThermoFisher) according to the manufacturer’s instructions. Purified *isoA* amplicons from enriched environmental samples were subjected to Illumina Mi-Seq sequencing by MrDNA (Shallowater, TX, USA) using an Illumina MiSeq platform.

*isoA* amplicon sequencing data were analysed using DADA2 pipeline [60] with default filtering parameters. Reads were truncated at 275 nucleotides and quality-filtered if their expected error was higher than two. After denoising the sequences using the estimated error rates, forward and reverse reads were merged. Resultant sequences were screened for chimeras and then manually checked by BLASTx [47]. Those OTUs with a top hit distinct from a ratified IsoA sequence were discarded, obtaining a final set of 134 unique OTUs for downstream analysis.

### Quantitative real-time PCR

Quantification of isoprene degraders in environmental samples was estimated by qPCR targeting the *isoA* gene using primers isoA14F and isoA511R (Additional file 1: Table S2). qPCR assays were carried out using a StepOne Plus real-time PCR instrument (Applied Biosystems). qPCR reactions (20 μl) contained 2–20 ng of DNA, 400 nM of each primer and 10 μl of SensiFast SYBR Hi-ROX kit (Bioline). The qPCR reaction consisted of an initial denaturation step at 95 °C for 3 min, followed by 40 cycles of 95 °C for 20 s, 60 °C for 20 s and 72 °C for 30 s. Data were acquired at 88 °C for 15 s to avoid quantification of primer dimers. Specificity of qPCR reactions was determined from melting curves obtained by increasing the temperature in 0.3 °C increments from 60 to 95 °C, followed by gel electrophoresis and clone library construction from several qPCR products.

The copy number of *isoA* genes was determined from qPCR of ten-fold dilution series (10^0^–10^8^ copies per μl) of DNA standards (Additional file 1: Figure S4). Standards were prepared by cloning the *isoA* gene of *Rhodococcus* sp. AD45 into the pGEM®T Easy vector (Promega) and using this as template DNA. The detection limit of the *isoA* qPCR assay was 10^2^ copies per 20 μl reaction.

Finally, *isoA* copies were normalised to 16S rRNA gene copy number in order to estimate the abundance of isoprene degraders in different environmental samples.

Number of copies of 16S rRNA genes was determined by qPCR using 519F and 907R primers [81]. Reactions (20 μl) contained 10–70 pg DNA, 400 nM of each primer and 10 μl of SensiFast SYBR Hi-ROX kit. The qPCR reaction consisted of an initial denaturation step at 95 °C for 3 min, followed by 40 cycles of 95 °C for 20 s, 55 °C for 20 s and 72 °C for 30 s. Data collection was performed at 72 °C for 15 s. Specificity of the qPCR reaction and quantification of 16S rRNA gene copy number were determined as above.

## Additional files

Additional file 1: **Table S1.** Sequences of hydroxylase α-subunits of soluble diiron monooxygenases used in the design of *isoA* primers. Table S2. Primers used in this study targeting the *isoA* gene. Table S3. Combinations of *isoA* primers tested in this study. Table S4. Control strains used in this study to validate the *isoA* gene primers isoA14F and isoA511R. Table S5. Environmental samples used in this study. Table S7. Alpha diversity of enriched environmental samples subjected to *isoA* amplicon sequencing. Figure S1. Alignment of IsoA sequences from representative isoprene-degrading bacteria and position of the new *isoA* primers. Figure S2. Preliminary validation of *isoA* primers. Figure S3. Diversity and abundance of Operational Taxonomic Units obtained by amplicon sequencing from enriched environmental samples. Figure S4. Calibration curve for *isoA* qPCR assays. Figure S5. Relative abundance of isoprene degraders in enriched environmental samples estimated by qPCR. (DOCX 920 kb)

Additional file 2: **Table S6.** Operational taxonomic units (OTUs) retrieved from enriched environmental samples targeting *isoA*. (XLSX 22 kb)
