# Supplementary material for: Gene probing reveals the widespread distribution, diversity and abundance of isoprene-degrading bacteria in the environment
Source: Microbiome. 2018 Dec 7;6:219. doi: 10.1186/s40168-018-0607-0 (PMC6286570; doi:10.1186/s40168-018-0607-0)
Supplement: Supplementary file 1 — Table S1. Sequences of hydroxylase α-subunits of soluble diiron monooxygenases used in the design of isoA primers. Table S2. Primers used in this study targeting the isoA gene. Table S3. Combinations of isoA primers tested in this study. Table S4. Control strains used in this study to validate the isoA gene primers isoA14F and isoA511R. Table S5. Environmental samples used in this study. Table S7. Alpha diversity of enriched environmental samples subjected to isoA amplicon sequencing. Figure S1. Alignment of IsoA sequences from representative isoprene-degrading bacteria and position of the new isoA primers. Figure S2. Preliminary validation of isoA primers. Figure S3. Diversity and abundance of Operational Taxonomic Units obtained by amplicon sequencing from enriched environmental samples. Figure S4. Calibration curve for isoA qPCR assays. Figure S5. Relative abundance of isoprene degraders in enriched environmental samples estimated by qPCR. (DOCX 920 kb) [file 40168_2018_607_MOESM1_ESM.docx]

**Additional File 1**

**Gene probing reveals the widespread distribution, diversity and abundance of isoprene-degrading bacteria in the environment.**

Ornella Carrión^1^, Nasmille L. Larke-Mejía^1^, Lisa Gibson^1^, Muhammad Farhan Ul Haque^1^, Javier Ramiro-García^2^, Terry J. McGenity^3^, J. Colin Murrell^1*^

^1^School of Environmental Sciences, University of East Anglia, Norwich, UK

^2^Luxembourg Centre for Systems Biomedicine, University of Luxembourg, Esch-sur-Alzette, Luxembourg

^3^School of Biological Sciences, University of Essex, Colchester, UK

^*^Corresponding authors:

J Colin Murrell, ^1^School of Environmental Sciences, University of East Anglia

Norwich Research Park, NR4 7TJ, UK

E-mail: [j.c.murrell@uea.ac.uk](mailto:j.c.murrell@uea.ac.uk)

Tel: (+44) 01603 592959

Fax: (+44) 01603 591327

Ornella Carrión, ^1^School of Environmental Sciences, University of East Anglia

Norwich Research Park, NR4 7TJ, UK

E-mail: [o.carrion-fonseca@uea.ac.uk](mailto:o.carrion-fonseca@uea.ac.uk)

Tel: (+44) 01603 592239

**Table S1. Sequences of hydroxylase α-subunits of soluble diiron monooxygenases used in the design of *isoA* primers.**

| **Microorganism** | **Enzyme** | **Genbank Accession Number** |
| --- | --- | --- |
| *Gordonia* sp. i37 | IsoMO | KU870746.1 |
| *Gordonia* sp. OPL2 | IsoMO | MK176353 |
| *Leifsonia* sp. i49 | IsoMO | KU870737.1 |
| *Loktanella* sp. i8b1 | IsoMO | KU870736.1 |
| *Micrococcus* sp. i61b | IsoMO | KU870739.1 |
| *Mycobacterium* sp. AT1 | IsoMO | KU870745.1 |
| *Mycobacterium* sp. i61a | IsoMO | KU870739.1 |
| *Nocardioides* sp. WS12 | IsoMO | MK176348 |
| *Rhodococcus* sp. ACPA1 | IsoMO | NSDX01000002.1 |
| *Rhodococcus* sp. ACPA4 | IsoMO | NZ_NSDY01000003.1 |
| *Rhodococcus* sp. ACS1 | IsoMO | NZ_NSDZ01000001.1 |
| *Rhodococcus* sp. ACS2 | IsoMO | MK176338 |
| *Rhodococcus* sp. AD45 | IsoMO | AJ249207.1 |
| *Rhodococcus* sp. i8a2 | IsoMO | KU870743.1 |
| *Rhodococcus* sp. i29a2 | IsoMO | KU870744.1 |
| *Rhodococcus* sp. LB1 | IsoMO | LTCZ01000014.1 |
| *Rhodococcus* sp. SC4 | IsoMO | LSBM01000309.1 |
| *Rhodococcus* sp. TD1 | IsoMO | MK176350 |
| *Rhodococcus* sp. TD2 | IsoMO | MK176351 |
| *Rhodococcus* sp. TD3 | IsoMO | MK176352 |
| *Rhodococcus* sp. WL1 | IsoMO | MK176349 |
| *Rhodococcus* sp. WS1 | IsoMO | MK176339 |
| *Rhodococcus* sp. WS2 | IsoMO | MK176340 |
| *Rhodococcus* sp. WS3 | IsoMO | MK176341 |
| *Rhodococcus* sp. WS4 | IsoMO | MK176342 |
| *Rhodococcus* sp. WS5 | IsoMO | MK176343 |
| *Rhodococcus* sp. WS6 | IsoMO | MK176344 |
| *Rhodococcus* sp. WS7 | IsoMO | MK176345 |
| *Rhodococcus* sp. WS10 | IsoMO | MK176347 |
| *Rhodococcus* sp. SK2ab | IsoMO | MK176356 |
| *Rhodococcus* sp. SK5 | IsoMO | MK176355 |
| *Rhodococcus erythropolis* i47 | IsoMO | KU870742.1 |
| *Rhodococcus opacus* PD630 | IsoMO | NZ_JH377098.1 |
| *Shinella* sp. i39 | IsoMO | KU870741.1 |
| *Sphingopyxis* sp. OPL5 | IsoMO | MK176354 |
| *Stappia* sp. iL42 | IsoMO | KU870740.1 |
| *Variovorax* sp. WS9 | IsoMO | MK176346 |
| *Variovorax* sp. WS11 | IsoMO | NZ_PXZZ01000003.1 |
| *Burkholderia cepacia* G4 | Toluene MO | AF349675 |
| *Gordonia* sp. TY5 | Propane MO | AB112920 |
| *Methylococcus capsulatus* Bath | Soluble methane MO | M90050 |
| *Methylosinus trichosporium* OB3b | Soluble methane MO | X55394 |
| *Mycobacterium* sp. M156 | Propene MO | AY455999 |
| *Mycobacterium chubuense* NBB4 | Ethene MO | GU174752 |
| *Mycobacterium chubuense* NBB4 | Propene MO | GU174753 |
| *Mycobacterium chubuense* NBB4 | Group 3 SDIMO | GU174751 |
| *Mycobacterium chubuense* NBB4 | Group 6 SDIMO | GU174750 |
| *Mycobacterium rhodosieae* JS60 | Ethane MO | AY243034 |
| *Nocardioides* sp. JS614 | Ethane MO | AY772007 |
| *Pseudomonas mendocina* KR1 | Toluene MO | M65106 |
| *Pseudonocardia* sp. K1 | Tetrohydrofuran MO | AJ296087 |
| *Rhodococcus rhodochrous* B-276 | Alkene MO | D37875 |
| *Thauera butanovora* | Butane MO | AY093933 |
| *Xanthobacter* sp. PY2 | Alkene MO | AJ012090 |

MO: monooxygenase. SDIMO: soluble diiron monooxygenase.

**Table S2. Primers used in this study targeting the *isoA* gene.**

| **Primer** | **Sequence (5’-3’)*** | **Nucleotide position respect to *isoA* from *Rhodococcus* sp. AD45** |
| --- | --- | --- |
| isoA14F | GVGACGAYTGGTAYGACA | 14 |
| isoA136F | TGGGABGAACCBTTCCGSGT | 136 |
| isoA300F | CATGGTCGARCABATGGC | 300 |
| isoA379F | GTBTTCGGVATGCTCGACGA | 379 |
| isoA511F | GTVAAGAAYTTCTTYGACGA | 511 |
| isoA511R | TCGTCRAAGAARTTCTTBAC | 511 |
| isoA862R | TCSAKCATGAAYTCCTTGAA | 862 |
| isoA1019R | GCRTTBGGBTTCCAGAACA | 1019 |

*Equimolar mixtures at degenerate positions: B (C, G, T); K (G, T); R (A, G); S (C, G); V (G,A,C); Y (C, T).

**Table S3. Combinations of *isoA* primers tested in this study.**

| **Combination** | **Primers** | **Amplicon size (bp)** | **Amplification from *Rhodococcus* sp. AD45 DNA** | **Amplification from *Variovorax* sp. WS9 DNA** | **Amplification from *Xanthobacter autotrophicus* Py2 DNA** |
| --- | --- | --- | --- | --- | --- |
| 1 | isoA14F  isoA511R | 497 | + | + | - |
| 2 | isoA14F  isoA862R | 848 | + | + | + |
| 3 | isoA14F  isoA1019R | 1005 | + | + | + |
| 4 | isoA136F  isoA511R | 375 | + | - | - |
| 5 | isoA136F  isoA862R | 726 | + | - | + |
| 6 | isoA136F  isoA1019R | 883 | + | + | - |
| 7 | isoA300F  isoA862R | 562 | - | + | - |
| 8 | isoA300F  isoA1019R | 719 | + | + | - |
| 9 | isoA379F  isoA862R | 483 | + | + | - |
| 10 | isoA379F  isoA1019R | 640 | + | + | - |
| 11 | isoA511F  isoA1019R | 485 | - | - | + |

**Table S4. Control strains used in this study to validate the *isoA* gene primers isoA14F and isoA511R**.

| **Strain** | **Control** | **Enzyme** | **Reference** | **Amplification with *iso*A primers** |
| --- | --- | --- | --- | --- |
| *Rhodococcus* sp. AD45 | Positive | Isoprene MO | [32] | + |
| *Rhodococcus* sp. WS1 | Positive | Isoprene MO | [38] | + |
| *Rhodococcus* sp. WS2 | Positive | Isoprene MO | [38] | + |
| *Rhodococcus* sp. WS3 | Positive | Isoprene MO | [38] | + |
| *Rhodococcus* sp. WS4 | Positive | Isoprene MO | [38] | + |
| *Rhodococcus* sp. WS5 | Positive | Isoprene MO | [38] | + |
| *Rhodococcus* sp. WS6 | Positive | Isoprene MO | [38] | + |
| *Rhodococcus* sp. WS7 | Positive | Isoprene MO | [38] | + |
| *Rhodococcus* sp. WS8 | Positive | Isoprene MO | [38] | + |
| *Rhodococcus* sp. WS10 | Positive | Isoprene MO | [38] | + |
| *Rhodococcus* sp. TD1 | Positive | Isoprene MO | [38] | + |
| *Rhodococcus* sp. TD2 | Positive | Isoprene MO | [38] | + |
| *Rhodococcus* sp. TD3 | Positive | Isoprene MO | [38] | + |
| *Rhodococcus* sp. WL1 | Positive | Isoprene MO | [38] | + |
| *Rhodococcus* sp. i47 | Positive | Isoprene MO | [83] | + |
| *Rhodococcus* sp. LB1 | Positive | Isoprene MO, Propane MO | [36] | + |
| *Rhodococcus* sp. SC4 | Positive | Isoprene MO, Propane MO | [36] | + |
| *Rhodococcus opacus* PD630 | Positive | Isoprene MO, Propane MO | [34] | + |
| *Rhodococcus* sp. ACPA1 | Positive | Isoprene MO | [35] | + |
| *Rhodococcus* sp. ACPA4 | Positive | Isoprene MO | [35] | + |
| *Rhodococcus* sp. ACS1 | Positive | Isoprene MO | [35] | + |
| *Rhodococcus* sp. ACS2 | Positive | Isoprene MO | Unpublished | + |
| *Rhodococcus* sp. SK2ab | Positive | Isoprene MO | Unpublished | + |
| *Rhodococcus* sp. SK5 | Positive | Isoprene MO | Unpublished | + |
| *Gordonia* sp. i37 | Positive | Isoprene MO, Propane MO | [31, 37] | + |
| *Gordonia* sp. OPL2 | Positive | Isoprene MO | [38] | + |
| *Nocardioides* sp. WS12 | Positive | Isoprene MO | [38] | + |
| *Variovorax* sp. WS9 | Positive | Isoprene MO | [38] | + |
| *Variovorax* sp. WS11 | Positive | Isoprene MO | [38] | + |
| *Sphingopyxis* sp. OPL5 | Positive | Isoprene MO | [38] | + |
| *Xanthobacter autotrophicus* Py2 | Negative | Alkene MO | [84] | - |
| *Methylococcus capsulatus* Bath | Negative | Methane MO | [85] | - |
| *Methylocella silvestris* BL2 | Negative | Methane MO, Propane MO | [86, 87] | - |
| *Mycobacterium* sp. NBB4 | Negative | Ethene MO, Propene MO, Group 3 SDIMO,  Group 6 SDIMO. | [88, 89] | - |
| *Pseudomonas mendocina* KR1 | Negative | Toluene MO | [90] | - |
| *Rhodococcus jostii* RHA1 | Negative | Propane MO | [91] | - |
| *Rhodococcus opacus* DSM 1069 | Negative | Unknown | [92] | - |
| *Rhodococcus rhodochrous* B276 | Negative | Alkene MO | [93] | - |
| *Rhodococcus rhodochrous* PNKb1 | Negative | Alkene MO | [94] | - |
| *Rhodococcus erythropolis* JCM 3201 | Negative | Alkane MO | [95] | - |
| *Pseudomonas putida* ML2 | Negative | Benzene dioxygenase | [96] | - |
| *Rhodococcus aetherivorans* I24 | Negative | Toluene dioxygenase | [97] | - |
| *Rhodococcus rhodochrous* DSM 43241 | Negative | Alkane MO | [98] | - |
| *Variovorax paradoxus* EPS | Negative | Alkanesulfonate MO | [55]; unpublished data | - |

MO: monooxygenase; SDIMO: soluble diiron monooxygenase.

| **Material** | **Type of sample** | **Sampling site** | **Location** | **Analysis** |
| --- | --- | --- | --- | --- |
| Ash leaves | Natural and enriched | University of East Anglia | Norwich, UK | Clone library  *iso*A amplicon sequencing  qPCR |
| Poplar leaves | Natural and enriched | University of East Anglia | Norwich, UK | *iso*A amplicon sequencing  qPCR |
| Willow leaves | Natural and enriched | University of East Anglia | Norwich, UK | Clone library  *iso*A amplicon sequencing  qPCR |
| Oil palm leaves A | Natural | Tawau | Sabah, Malaysia | qPCR |
| Oil palm leaves B | Natural and enriched | Sepang | Selangor, Malaysia | Clone library  *isoA* amplicon sequencing  *qPCR* |
| Oil palm leaves C | Enriched | Kew Gardens | London, UK | Clone library  *iso*A amplicon sequencing |
| Oil palm soil A | Natural | Sepang | Selangor, Malaysia | qPCR |
| Oil palm soil B | Natural | Sepang | Selangor, Malaysia | qPCR |
| Oil palm soil C | Natural | Sepang | Selangor, Malaysia | qPCR |
| Oil palm soil D | Enriched | Kew Gardens | London, UK | *iso*A amplicon sequencing |
| Willow soil | Natural and enriched | University of East Anglia | Norwich, UK | Clone library  *isoA* amplicon sequencing  qPCR |
| Tyre dump soil | Natural and enriched | Industrial park | Fakenham, UK | Clone library  *iso*A amplicon sequencing  qPCR |
| Landfill soil | Natural | Landfill | Strumpshaw, UK | qPCR |
| Grassland soil A | Natural | Bowthorpe | Norwich, UK | qPCR |
| Grassland soil B | Natural | University of East Anglia | Norwich, UK | qPCR |
| Coastal sediment | Natural and enriched | Penarth beach | Penarth, UK | Clone library  *isoA* amplicon sequencing  qPCR |
| Salt marsh sediment A | Natural and enriched | Stiffkey salt marsh | Stiffkey, UK | Clone library  *isoA* amplicon sequencing  qPCR |
| Salt marsh sediment B | Natural | Warham salt marsh | Warham, UK | qPCR |
| Freshwater sediment | Natural and enriched | University of East Anglia lake | Norwich, UK | Clone library  *isoA* amplicon sequencing  qPCR |

**Table S5. Environmental samples used in this study.**

**Table S7. Alpha diversity of enriched environmental samples subjected to *isoA* amplicon sequencing.** Shannon index for each enrichment was calculated using the packages phyloseq [99], ggplot2 [100] and tidyverse [101] included in R 3.4.4 [102].

| **Enriched environmental sample** | **Shannon index** |
| --- | --- |
| Ash leaves | 1.65 |
| Willow leaves | 1.05 |
| Poplar leaves | 1.49 |
| Oil palm leaves B | 0.25 |
| Oil palm leaves C | 1.19 |
| Oil palm soil D | 0.63 |
| Willow soil | 1.83 |
| Tyre dump soil | 2.11 |
| Freshwater sediment | 1.14 |
| Coastal sediment | 0.43 |
| Salt marsh sediment A | 0.18 |


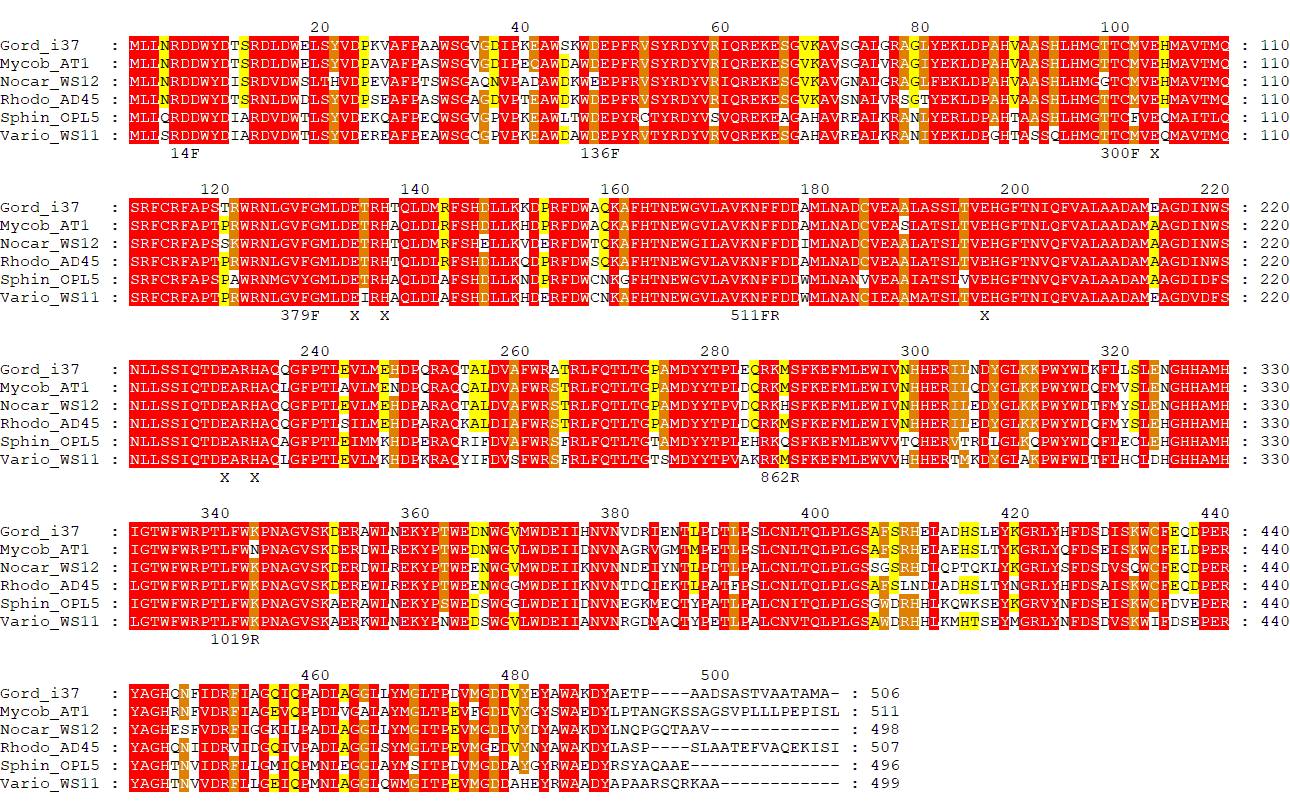


**Figure S1. Alignment of IsoA sequences from representative isoprene-degrading bacteria and position of the new *isoA* primers.**  Alignment of IsoA sequences was done using the ClustalW package included in BioEdit Sequence Alignment Editor v7.2.6 [103]. Conserved domains were analysed using GeneDoc v2.5.010 [104]. Residues with identical or similar properties are highlighted in red, orange or yellow if they are conserved in all six, at least five or at least four polypeptides. The positions of iron binding ligands are marked with an “X” below. Start positions of the new *isoA* primers are indicated as follows: 14F: isoA14F; 136F: isoA136F; 300F: isoA300F; 379F: isoA379F; 511FR: isoA511F and isoA511R; 862R: isoA862R; 1019R3: isoA1019R. Strain names are: *Gordonia* sp. i37, *Mycobacterium* sp. AT1; *Nocardioides* sp. WS12; *Rhodococcus* sp. AD45; *Sphingopyxis* OPL5, and *Variovorax* sp. WS11. Accession numbers of these sequences are listed in Additional File 1; Table S1.


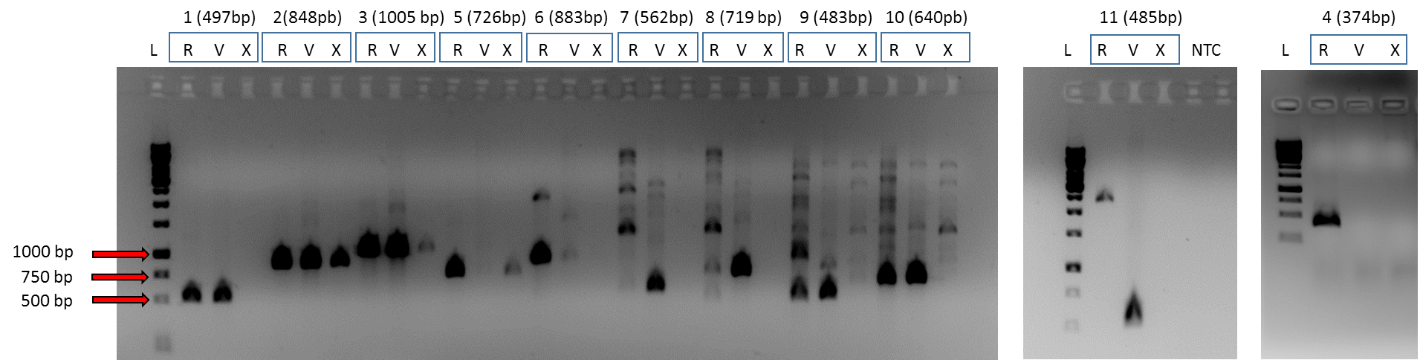


**Figure S2. Preliminary validation of *isoA* primers.** Eleven combinations of PCR primers were tested with genomic DNA from isoprene-degrading bacteria *Rhodococcus* sp. AD45 (R) and *Variovorax* sp. WS9 (V). Genomic DNA from *Xanthobacter autotrophicus* Py2 (X) was included as a negative control. Combination of primers and expected PCR amplicon size are indicated above gel images. Combination 1 was selected for further validation on more positive and negative control strains and environmental samples. L: GeneRuler^TM^ 1kb Plus Ladder (ThermoFisher); NTC: non-template control.


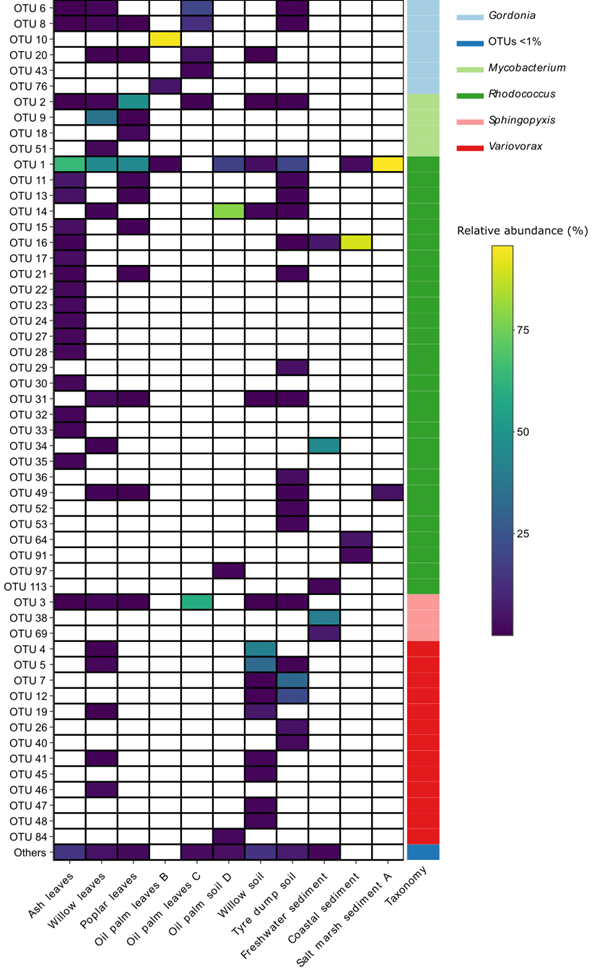


**Figure S3. Diversity and abundance of Operational Taxonomic Units obtained by amplicon sequencing from enriched environmental samples.** Analysis of *isoA* sequences from enriched environmental samples with the DADA2 pipeline [60] yielded a final set of 134 Operational Taxonomic Units (OTUs). Only OTUs with ≥1% relative abundance in at least one sample are represented. Taxonomy column indicates the genus of bona-fide isoprene degraders phylogenetically closer to a particular OTU. The heatmap was constructed using the packages plotly [105], heatmaply [106], ggplot2 [100] and tidyverse [101] included in R 3.4.4 [102].


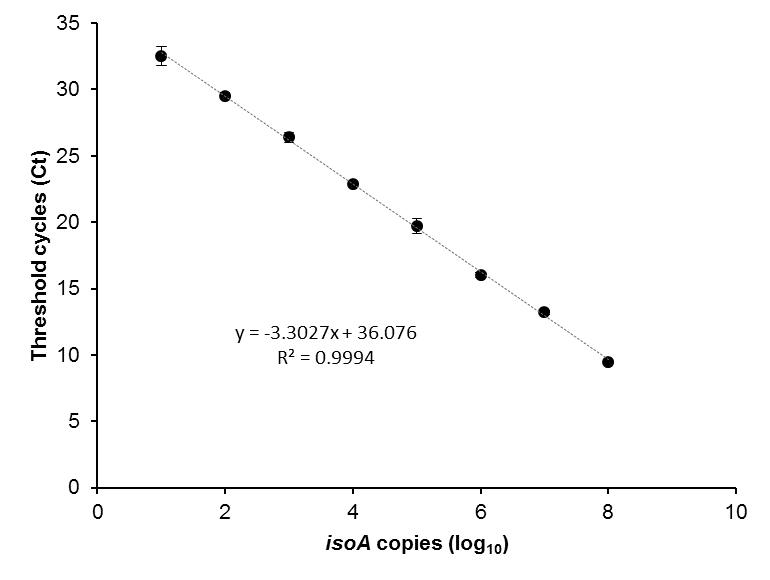


**Figure S4. Calibration curve for *isoA* qPCR assays.** Ten-fold dilutions of standards were used as template for qPCR of *isoA* genes. Standards were prepared by cloning the *isoA* gene from *Rhodococcus* sp. AD45 into pGEM®T Easy vector (Promega). Threshold cycle (Ct) values were plotted against known copy numbers of the standards. Each point represents the average of triplicate samples with error bars (smaller than marker if not visible) showing the respective standard deviations.


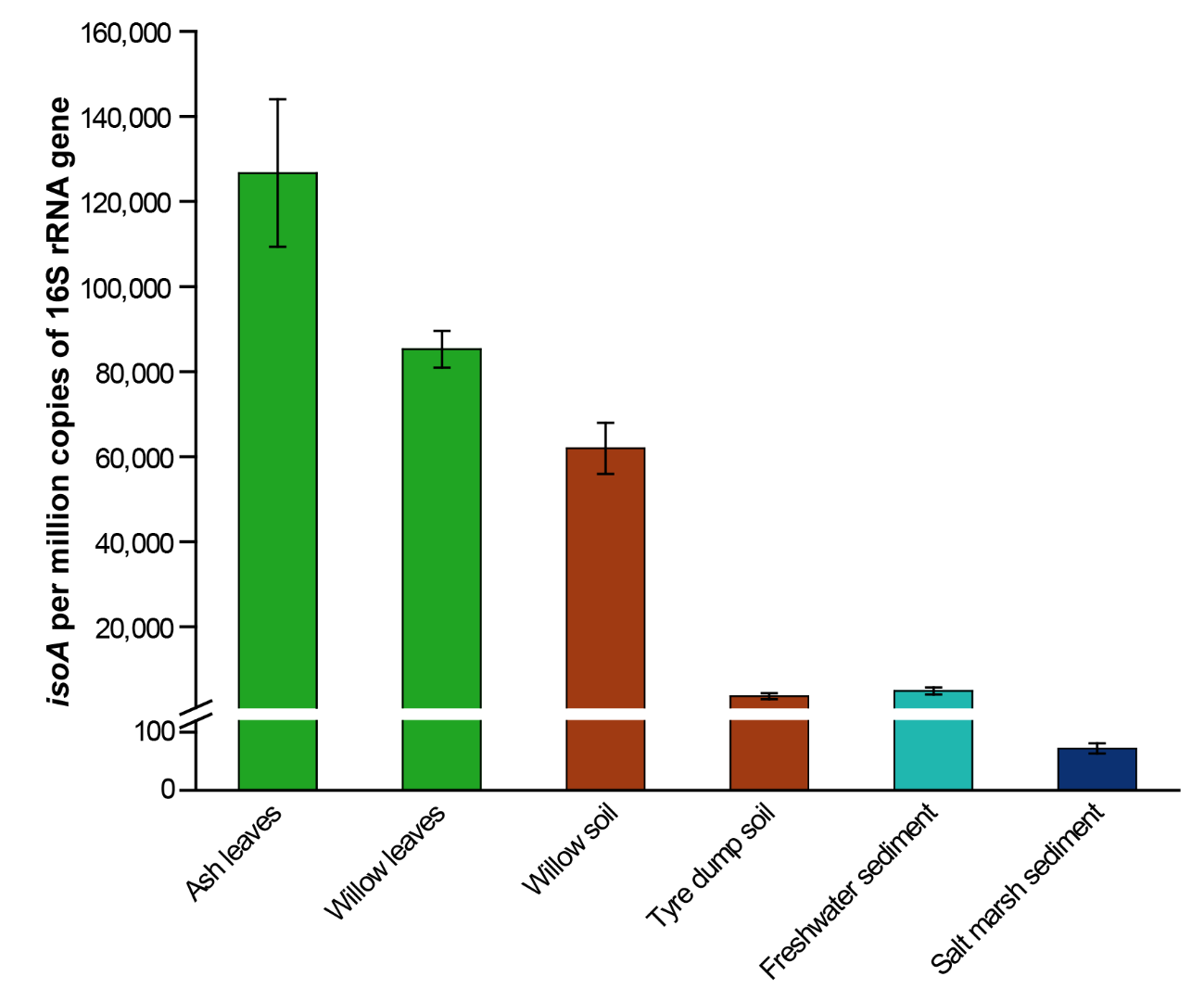


**Figure S5. Relative abundance of isoprene degraders in enriched environmental samples estimated by qPCR.** Number of *isoA* genes are normalised to copies of 16S rRNA gene in each sample. Results shown are the average of triplicate samples. Error bars represent standard deviations. Leaf samples are represented in green, soils in brown, freshwater environments in light blue and marine sediments in dark blue.

**References**

83. Johnston A. Molecular ecology of marine isoprene degradation. PhD thesis. School of Environmental Sciences, University of East Anglia, Norwich, UK. 2014.

1. Small FJ, Ensign SA. Alkene monooxygenase from *Xanthobacter* strain Py2. J Biol Chem. 1997;272:24913–20.
2. Stainthorpe AC, Lees V, Salmond, GPC, Dalton H, Murrell JC. The methane monooxygenase gene cluster of *Methylococcus capsulatus* (Bath). Gene. 1990;91:27–34.
3. Chen Y, Scanlan J, Song L, Crombie AT, Rahman MT, Schäfer H *et al*. γ-glutamylmethylamide is an essential intermediate in the metabolism of methylamine by *Methylocella silvestris*. Appl Environ Microbiol. 2010;76:4530-7.
4. Crombie AT, Murrell JC. Trace-gas metabolic versatility of the facultative methanotroph *Methylocella silvestris*. Nature. 2014;510:148–51.
5. Coleman NV, Yau S, Wilson NL, Nolan LM, Migocki MD, Ly MA *et al.* Untangling the multiple monooxygenases of *Mycobacterium chubuense* strain NBB4, a versatile hydrocarbon degrader. Environ Microbiol Rep. 2011;3:297–307.
6. Martin KE, Ozsvar J, Coleman NV. SmoXYB1C1Z of *Mycobacterium* sp. strain NBB4: A soluble methane monooxygenase (sMMO)-like enzyme, active on C_2_ to C_4_ alkanes and alkenes. Appl Environ Microbiol. 2014;80:5801-6.
7. Whited GM, Gibson DT. Toluene-4-monooxygenase, a three-component enzyme system that catalyzes the oxidation of toluene to p-cresol in *Pseudomonas mendocina* KR1. J Bacteriol. 1991;173:3010–16.
8. Sharp JO, Sales CM, LeBlanc JC, Liu J, Wood TK, Eltis LD *et al.* An inducible propane monooxygenase is responsible for N-nitrosodimethylamine degradation by *Rhodococcus* sp. strain RHA1. Appl Environ Microbiol. 2007;73:6930–8.
9. Eggeling L, Sahm H. Degradation of coniferyl alcohol and other lignin-related aromatic compounds by *Nocardia* sp. DSM 1069. Arch Microbiol. 1980;126:141-8.
10. Furuhashi K, Taoka A, Uchida S, Karube I, Suzuki S. Production of 1,2-epoxyalkanes from 1-alkenes by *Nocardia corallina* B-276. European J Appl Microbiol Biotechnol. 1981;12:39–45.
11. Woods NR, Murrell JC. The metabolism of propane in *Rhodococcus rhodochrous* PNKb1. J Gen Microbiol. 1989;135:2335-44.
12. Táncsics A, Benedek T, Szoboszlay S, Veres PG, Farkas M, Máthé I *et al.* The detection and phylogenetic analysis of the alkane 1-monooxygenase gene of members of the genus *Rhodococcus*. Syst Appl Microbiol. 2015;38:1–7.
13. Tan HM, Mason JR. Cloning and expression of the plasmid-encoded benzene dioxygenase genes from *Pseudomonas putida* ML2. FEMS Microbiol Lett. 1990;72: 259–64.
14. Chartrain M, Jackey B, Taylor C, Sandford V, Gbewonyo K, Lister L *et al.* Bioconversion of indene to *cis*-(1S,2R)-indandiol and *trans*-(1R,2R)-indandiol by *Rhodococcus* species. J Ferment Bioeng. 1998;86:550–8.
15. Vomberg A, Klinner U. Distribution of *alk*B genes within n-alkane-degrading bacteria. J Appl Microbiol. 2000;89:339–48.
16. McMurdie PJ, Holmes S. phyloseq: An R package for reproducible interactive analysis and graphics of microbiome census data. PLoS ONE. 2013;8:e61217.
17. Wickham H. ggplot2: Elegant graphics for data analysis. New York: Springer-Verlag; 2016.
18. Wickham H. tidyverse: Easily installed and load the “tidyverse”. 2017. https://CRAN.R-project.org/package=tidyverse. Accessed 11 Sept 2018.
19. R Core Team. R: A language and environment for statistical computing. R foundation for statistical computing, Vienna, Austria. 2018. https://www.R-project.org. Accessed 11 Sept 2018.

Hall TA. BioEdit: a user-friendly biological sequence alignment editor and analysis program for Windows 95/98/NT. Nucl Acids Symp Ser. 1999;41:95-8.

1. Nicholas KB, Nicholas HBJ. Genedoc: a tool for editing and annotating multiple sequence alignments. Distributed by the author. 1997.
2. Sievert C. plotly for R. 2018. https://plotly-book.cpsievert.me. Accessed 11 Sept 2018.
3. Galili T, O’Callaghan A, Sidi J, Sievert C. heatmaply: an R package for creating interactive cluster heatmaps for online publishing. Bioinformatics. 2018;34:1600-2.
